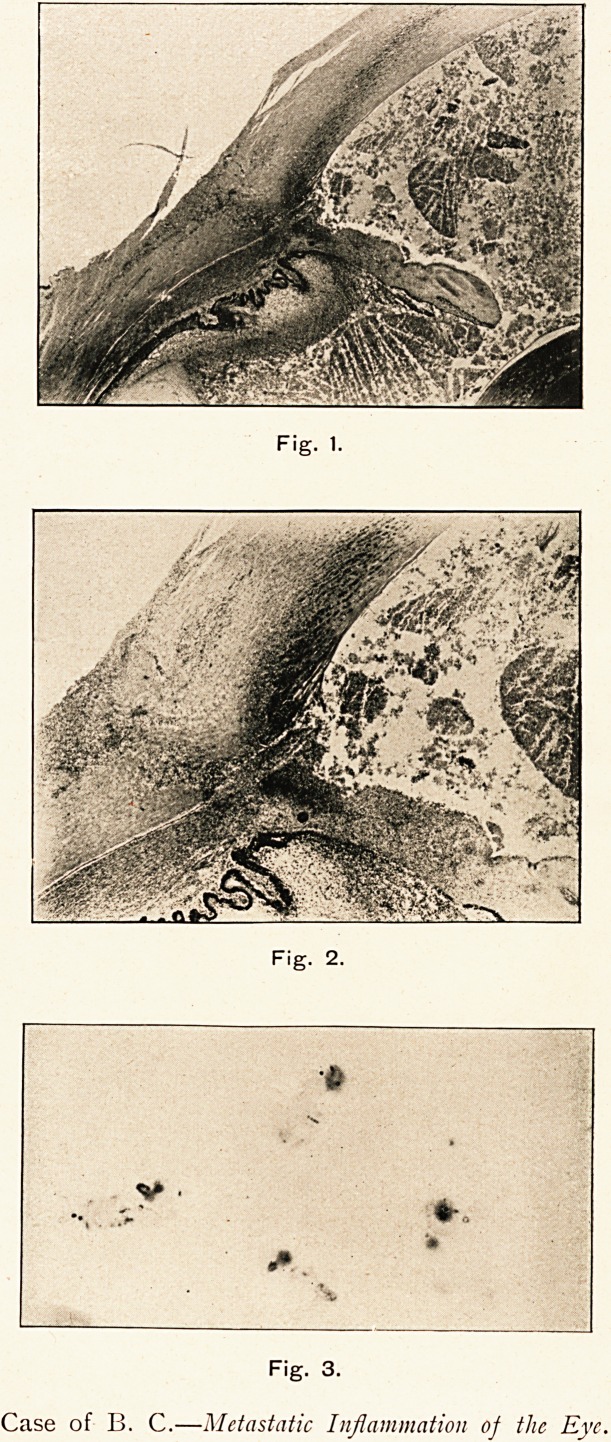# The Long Fox Lecture: Metastatic Inflammations of the Eye

**Published:** 1909-03

**Authors:** J. Herbert Parsons

**Affiliations:** Associate of University College, Bristol; Assistant Ophthalmic Surgeon, University College Hospital, London; Assistant Surgeon, Royal London (Moorfields) Ophthalmic Hospital; Ophthalmic Surgeon, Hospital for Sick Children, Great Ormond Street, London


*" Scire est nescire, nisi id me*
*Scire alius sciret."*

MARCH, 1909.

INDEX THROUGH

### THE LONG FOX LECTURE:

THE FIFTH ANNUAL LECTURE ARRANGED BY THE COMMITTEE OF THE LONG FOX MEMORIAL,
DELIVERED IN THE MEDICAL LIBRARY, UNIVERSITY COLLEGE, BRISTOL, ON NOVEMBER 5TH, 1908.

F. RICHARDSON CROSS, F.R.C.S., *in the Chair.*

BY

J. HERBERT PARSONS, B.S., D.SC. (Lond.), F.R.C.S. (Eng.),
*Associate of University College, Bristol;*
*Assistant Ophthalmic Surgeon, University College Hospital, London;*
*Assistant Surgeon, Royal London (Moorfields) Ophthalmic Hospital;*
*Ophthalmic Surgeon, Hospital for Sick Children, Great Ormond Street, London.*

ON

### METASTATIC INFLAMMATIONS OF THE EYE.

---

MY first duty is the pleasant one of thanking you for the honour you have done me in electing me to give the fifth lecture in this series. My personal acquaintance with Dr. Long Fox was slight, but it appeared to me, even in those early days, that no one could fail to be struck with his geniality, his intellectual superiority, and the high purpose which ruled his life. It is not surprising that you should desire to perpetuate his beneficent influence, and those who are called upon to deliver this lecture cannot fail to be stimulated to approach as near as may be to his ideal, and thus fulfil your intentions.

I have chosen for my subject "Metastatic or Endogenous Inflammations of the Eye," by which I mean inflammations of the eye due to bacteria or toxins which are derived from a source in some other part of the body. It will be at once appreciated that the subject is very wide in its scope, and includes a number of well-known diseases of the eye. Time will not permit me to review these exhaustively, and I shall use them only as a safe starting-point, and for the illustrations they supply of important general deductions which may be reasonably accepted. I do not propose to include (under the term *metastatic*) transmission by direct continuity, though in cases where the meninges of the brain are affected it is not always easy to eliminate this mode. Two other paths are open, by the lymph and blood streams. Of these, the former is rare, and in most cases we shall have to deal with blood transmission.

It will be convenient to take ocular tuberculosis as a typical form of metastatic inflammation. Clinically it can generally be recognised with certainty, and the diagnosis is usually placed beyond doubt if microscopic examination is feasible. Tubercle almost invariably attacks some part of the uveal tract, either the iris, ciliary body or choroid. It is probable that the retina is never affected primarily, and only a few cases of tubercle of the optic nerve head are on record. One such case came under my care at Great Ormond Street Children's Hospital, and has been reported by Mr. Coats.[1] This peculiarity of anatomical distribution introduces one of the main points upon which I wish to lay stress, viz. *the selectivity of site in metastatic inflammation of the eye.*

As regards tubercle of the iris, there is the possibility of direct transmission from the cornea or conjunctiva. Some time ago I inoculated the corneæ of rabbits with a virulent culture of tubercle bacilli. In no case was tubercle of the iris caused unless the anterior chamber was penetrated. Indeed, tubercle of the cornea is produced with extreme difficulty experimentally, and primary tubercle of the cornea is excessively rare clinically. Much discussion has arisen as to whether tubercle of the iris is primary or secondary. The matter need not detain us now, since it matters little for the aspect from which we are considering the subject whether the iridic is the first focus of deposition or a secondary one ; in either case the inflammation is endogenous in origin and due to blood transmission.

A very striking example of the selective action of organisms on the tissues is found in gonorrhœa. Gonorrhœal iritis is much commoner than was at one time supposed. It may occur during the acute attack of urethritis, the exudates into the anterior chamber having then a peculiar gelatinous appearance, or it occurs as a form of recurrent iritis. In the latter variety it seldom supervenes until after an attack of arthritis, usually in the knees, and it is nearly always associated with gonorrhœal "rheumatism." Thus, gonorrhœal rheumatism and iritis are both manifestations of metastatic deposits of gonococci in the tissues of distant organs. It is well known that gonococci remain virulent in the body for a long time after the acute attack. In the recurrent form of gonorrhœal iritis it is probable that they become encapsuled, lying quiescent for months or years and wakening into activity as the result of trauma or intercurrent inflammation of a different origin. An interesting experiment by Stock[2] may be mentioned in this connection. He found that the transference of a completely healed tuberculous iris from a blood infection in a rabbit to a second rabbit produced severe tuberculosis. As might be anticipated from their anatomical and physiological continuity and similarity, inflammation of one part of the uveal tract is very likely to spread to another. Indeed, it is probably rare for one part to be affected alone. Thus an iritis is nearly always, if not always, accompanied by some cyclitis, and *vice versa.* Often the whole uveal tract seems to suffer simultaneously and equally. Cyclitis as an accompaniment of choroiditis is probably more frequent than we have been accustomed to think. It is somewhat remarkable, therefore, that gonorrhœal iritis rarely spreads in any great degree to the ciliary body, and that the choroid is almost never affected. This shows a striking selective influence of the various tissues by the organisms. It may be explained by supposing, in the terms of Ehrlich's theory, that certain tissues are particularly rich in appropriate receptors for specific organisms, and that these receptors may be completely absent in other tissues.

The same selection of the iris was shown by an extremely virulent strain of streptococci in a case of septicæmia which came under my observation at Great Ormond Street. The following is a brief abstract of the notes :—

Beatrice C. was admitted to the Children's Hospital on July 14th, 1908, under the care of Mr. Fairbank, with a temperature of 103°. There had been a discharge from the left ear for four years. On admission there was a fluctuating swelling over the left mastoid. On the same day Mr. Fairbank did a complete mastoid operation. The antrum was full of pus. The lateral sinus was exposed but not opened. The abscess was drained through a counter opening in the posterior triangle. The temperature continued high and irregular. On July 18th, the mastoid was cut away posteriorly, the internal jugular vein ligatured, and the lateral sinus opened and packed with gauze. It was not thrombosed. The temperature continued remittent, rising to 103°. The left optic disc appeared slightly blurred. On July 23rd cultures were taken from the blood ; they showed the presence of pure streptococci. More bone was removed posteriorly, but nothing was discovered. On July 24th the lateral sinus was opened up and washed through from the jugular vein, a considerable quantity of pus being evacuated. The eyes had been repeatedly examined, but no definite neuritis could be made out. On this day several flakes of lymph were seen upon the left iris. On July 25th the cornea was steamy, there was intense iritis with much ciliary injection. The fundus could not be seen. Atropine was ordered. The right disc looked normal. There was no proptosis of the left eye. The left side of the body was paretic. The skull was trephined, and the cerebrum and cerebellum both explored, but only clear fluid was obtained. Examination of cerebro-spinal fluid showed equal numbers of lymphocytes and polymorphonuclear leucocytes : it was sterile. Cultures from the mastoid wound showed staphylococcus aureus. On July 26th the left eye was still more inflamed and the conjunctiva was chemosed. The child was becoming unconscious, and nasal feeding was commenced. On July 27th the left cornea was like ground glass. The lids were separated owing to the chemosis, but there was no proptosis. The right disc looked normal, but

Fig. 1.

Fig. 2.

Fig. 3.

Case of B. C.—*Metastatic Inflammation of the Eye.*

there were punctate hemorrhages in the retina above the disc. On July 28th the legs and arms, up to now flaccid, became more rigid. Urine and fæces were voided involuntarily. The right disc was blurred, but there was not much swelling. On July 29th the child was comatose; temperature 105–6°; respirations 46. Some sloughing of scalp in right temporal region. Right optic neuritis marked, and many retinal hemorrhages, distributed along the vessels. The patient died on July 31st.

*Post-mortem Examination.*—The left jugular vein contained septic thrombus as high as the horizontal part of the lateral sinus. The left inferior petrosal sinus contained septic clot. The left superior petrosal and cavernous sinuses were not thrombosed. All the sinuses on the right side were normal. There was a small abscess in the *right* uncus. Right temporo-sphenoidal lobe softened Brain congested, no meningitis. No thrombosis of cerebral veins. There was a small broken-down septic infarct in the upper lobe of the right lung. Heart, prominent vegetations on the mitral valve. Left kidney, enormous infarct involving the whole of the upper part. Liver, fatty. Spleen, slightly enlarged (it was easily palpable when the child was admitted).

The eyes were hardened in 10 per cent. formol.

I am indebted to Mr. G. Coats for kindly preparing sections and photographs. (Figs. 1 to 3.)

*Left Eye.*—Cornea necrotic. It is denuded of epithelium. possible largely a *post-mortem* effect. Bowman's membrane is intact. The substantia propria is infiltrated with polymorphonuclear leucocytes, which fill the interlamellar spaces of the periphery. Towards the centre of the cornea only the anterior spaces are infiltrated, and the amount of infiltration diminishes from the periphery towards the centre. In the posterior part of the centre of the cornea there are no stained nuclei, so that we may conclude that the corneal corpuscles are dead, and that the greater part of the cornea is necrosed. The infiltration at the periphery is densest around the canal of Schlemm. Here the corneo-scleral structures are entirely masked by the deeply-stained nuclei of the polymorphonuclear leucocytes. (Fig. 1.)

The iris is necrotic. At the periphery it is densely infiltrated with leucocytes. The inner half towards the pupil is little infiltrated, but the nuclei of the stroma cells are unstained. The posterior surface of the iris is becoming denuded of the retinal pigment epithelium. (Fig. 2.)

The anterior chamber is filled with a coagulum which contains an enormous number of leucocytes in various stages of degeneration. Patches show scarcely any nuclear staining, whilst other patches stain feebly. The angles of the chamber are filled with well-stained leucocytes.

The ciliary body is intensely inflamed but not necrotic, for the nuclei of its structural cells stain well. It is covered with a purulent exudation which pervades the vitreous. Throughout the vitreous the exudation is composed of a fibrinous network. Leucocytes are sparse everywhere except around the circle of the ciliary body, where they stain fairly well. The retinal epithelium covering the ciliary processes shows signs of desquamation. A considerable part of the suspensory ligament has disappeared, the fibres having been absorbed. The broken ends of the fibres are seen, their attachments to the ciliary body being intact.

The lens is commencing to become cataractous, the peripheral fibres, chiefly at the equator and posterior pole, being broken up into globules.

The retina and choroid show remarkably little infiltration, though the former is much congested.

The optic nerve is œdematous, but not infiltrated at all, and the arachnoid of the vaginal space is very little infiltrated, and that only near the eye. Posteriorly the nerve and its coverings look quite normal.

Sections stained for micro-organisms showed the presence of large numbers of streptococci. (Fig. 3.) These were most numerous, and best stained in the neighbourhood of the periphery of the iris, and in the angles of the anterior chamber. Streptococci were present in the exudation on the ciliary body, but here they were scanty, and showed signs of degeneration by staining feebly. In the posterior part of the eye no organisms were seen.

*Right Eye.*—The anterior part of the right eye is normal. The optic nerve head is swollen and œdematous, without much infiltration. It thus shows the signs of papillœdema or choked disc, rather than those of a true optic neuritis. It is probable that the swelling is due rather to intracranial pressure than to infective inflammation. The condition of the left disc confirms this view. It must be stated, however, that the arachnoid of the right optic nerve shows more signs of infiltration, extending farther back along the nerve, than that of the left, and the vaginal space is more dilated.

It will be seen that in this case the primary seat of metastasis was undoubtedly the iris. Metastatic iritis is not uncommon in experimental intravenous injection of streptococci in animals, but bacterial emboli in other parts of the uveal tract are also frequently found. We may bear in mind here the cases of so-called "rheumatic iritis" obtained in rabbits occasionally by Poynton and Paine[3] after intravenous injection of the diplococcus rheumaticus. This iritis resembles in every respect a septic iritis in its intense virulence, and does not in the slightest degree resemble the iritis which we are clinically accustomed loosely to call rheumatic.

Tubercle affects the choroid in two forms, miliary and conglomerate or solitary. The former is an almost constant accompaniment of the late stages of acute general tuberculosis and of tuberculous meningitis. In the last-mentioned disease, though miliary tubercles are often found *post-mortem* studding the sheath of the optic nerve, the choroidal affection must be regarded as a true metastasis. The posterior part of the nerve is always most involved, and no continuity with the choroidal disease can be demonstrated. Moreover, in most cases the nerve and its sheaths are free from deposits. The same fact is true of endophthalmitis occurring in the course of pneumococcic meningitis, as shown by Axenfeld,[4] and in a case of traumatic streptococcic meningitis reported by de Lieto Vollaro.[5] According to Axenfeld, there is no proved case of transmission by the nerve sheath as suggested in certain cases (Saltini, Silcock, Treacher Collins,[6] and others).

Conglomerate tubercle of the choroid is of particular importance in the clinical diagnosis of metastatic endophthalmitis. It is not very uncommon to meet with cases in which in some part of the fundus a billowy white mass of exudate obscures the normal structures. It has been customary, though on insufficient grounds, to regard these as cases of conglomerate tubercle, which they much resemble in ophthalmoscopic appearances. The condition, however, not infrequently occurs in adults, and, unlike tubercle, gradually subsides, leaving a scarred area behind. It is true that undoubted tubercle occasionally, though rarely, follows a similar course, as has been shown notably in two cases reported by Jessop.[7] In a somewhat allied group of cases an area of retina is separated from the choroid by exudate ; the irregularly prominent retina is œdematous and white, and the retinal vessels in the neighbourhood, especially distal to the area, are often profoundly diseased. One of these cases, under the care of Mr. Marcus Gunn, was reported by Paton[8] as a case of doubtful tubercle. At a later stage I had the opportunity of examining it microscopically.[9] It was possible to state definitely that it was not tuberculous, but the exact nature was somewhat problematic. Recently Coats has examined microscopically several such cases, and has collected others from the literature. They will shortly be published in the

*Royal London Ophthalmic Hospital Reports.*[10] He favours the view that they are due to subretinal hemorrhage, and I have little doubt that this is the correct explanation in many cases. There is reason, however, to think that other such cases, distinguished from them with difficulty clinically or not at all, are due to metastatic inflammation of a low grade of virulence.

In the condition described by Roth[11] as retinitis septica, white oval spots of considerable size, with or without hemorrhages, occur in the retina, usually not far from the disc. Such spots may be seen in anæmia, leukæmia, diabetes, &c., but are seen in their characteristic form in septicæmic conditions. It is quite certain that they occur in some cases of true metastatic retinitis, as recorded by Axenfeld and Goh,[12] and by Grunert.[13] Axenfeld and Goh's case is particularly important. The patient died. One eye had gone on to suppurative panophthalmitis, and was lost; the other, less affected, contained inflammatory nodules in the retina and choroid. These deposits contained masses of pneumococci, which showed degenerative changes, staining badly, and in other retinal patches had disappeared. Grunert and v. Michel found masses of streptococci in similar patches. Cases of staphylococcic infection leading to changes in the fundus with spontaneous resolution are reported by Holmes Spicer[14] and Schanz.[15] Spicer's cases showed the variability of results from a similar cause. In one there was a retinal abscess, in another retinal phlebitis, in another a local detachment of retina, all due to staphylococcic metastasis from boils. He also recorded a case of retinal phlebitis and local keratitis profunda, due to ptomaine poisoning. Probably a case which I saw, and which has been published by Pernet,[16] comes under the same category. The woman, aged 28, had acquired syphilis. During the secondary stage she complained of bad sight. The retinæ were œdematous, and the vitreous of each eye full of exudates. There was a yellowish reflex, and an hypopyon appeared in the right eye. After prolonged treatment with anti-syphilitic remedies, the vision in each eye bcame almost normal. In this case it is certain that the ocular inflammation was endogenous, and it is unlikely that it was due to syphilis uncomplicated by a septic process.

The pneumococcic cases emphasise another feature of metastatic inflammation of the eye upon which I wish to lay special stress, viz. *the strong tendency for endogenous bacterial metastasis to undergo spontaneous resolution.* We know that pneumococci are amongst the most dangerous organisms which attack the eye. They are responsible for hypopyon ulcer, and for a large number of cases of panophthalmitis, notably those following intraocular operation in the presence of a lacrymal mucocele. Thus, if virulent pneumococci are introduced into the vitreous from outside the body, suppurative retinitis rapidly supervenes, spreads to other parts of the eye, and the eye is inevitably lost from panophthalmitis. On the other hand, pathogenic organisms introduced into the blood stream encounter adverse circumstances which lead to their obliteration or attenuation. They have to fight against the normal protective mechanism, which is actually reinforced by virtue of their presence. Hence, though the organisms may be victors in the long run, they have to run the gauntlet of fierce opposition. In the tissues they have to fight another battle with the local protective mechanism. I have already laid stress upon the predilection of given organisms for specific tissues. It may be that this is merely a ruse of the protective mechanism. Possibly only those tissues which contain the specific receptors for the given organism possess the capacity for manufacturing the corresponding anti-bodies. However that may be, it is certain that virulent organisms deposited in the eye by the blood stream become attenuated and liable to die out. Hence we must not conclude that because spontaneous inflammation tends to resolve itself it must necessarily have been due to toxins, and that bacteria are not present. We have every reason to believe now that virulent pyogenic organisms, brought to the eye by the blood stream, may fail to cause suppuration, and merely set up a sub-acute or chronic plastic inflammation. It is clear that this fact is one of very great significance.

I have instanced the predilection of the tubercle bacillus for the uveal tract. Other organisms, such as the pneumococcus, staphylococcus, often the streptococcus, &c., show a preference for the retina. This is particularly so when, as is often the case, both eyes are affected. In unilateral metastasis embolic affection of the uvea is frequent. The prevalence of retinal infection cannot be accounted for by special narrowness of the capillaries, for those of the uveal tract are equally small (Sattler). Moreover, in rabbits there is no predisposition for the vascular part of the retina to be specially affected. Stock always obtained uveal metastases, never retinal, from intravenous injections of bacillus pyocyaneus in rabbits. Even with tubercle he found that virulent cultures oftener produced slight lesions in the choroid than in the iris or ciliary body. He attributed this to encapsulation of the bacilli. It is interesting to note that previous injury to the eye in rabbits and cats by cauterisation, foreign body, &c., increases the tendency to metastatic deposits with organisms of slight virulence.

Metastatic ophthalmia was far commoner in the days when puerperal fever and pyæmia were rife, and was much more frequent in the former than in the latter disease. In both streptococci are usually the cause, but the streptococci of puerperal fever seem to have a special affinity for the eye (Axenfeld). Cryptogenetic cases, or those in which a primary focus cannot be discovered, are probably often due to lesions in the gastro-intestinal tract (cf. Holmes Spicer). This is shown by the fact that typhoid bacilli and bacterium coli have been found. Pneumococci are much commoner in cryptogenetic cases than in puerperal or pyæmic. The meningococcus intracellularis of Weichselbaum has been found several times. The organism disappears quickly from the eye, so that it is probably not infrequently missed.

One example of undoubted metastatic inflammation of the eye is found in so-called pseudoglioma. The condition is thus designated because of the resemblance to glioma retinæ ; indeed, they may be indistinguishable clinically. Pseudoglioma may be due to a congenital malformation, to conglomerate tubercle, or to metastatic iridocyclitis. In the last form there is often the history of convulsions, acute specific fever, or some other illness. Often the ocular inflammation produces so little outward manifestation that it is noticed only long after the illness, or there may be no history of illness at all. The child is usually brought for examination for the same reason as in glioma, viz. a yellow reflex through the pupil (amaurotic cat's eye). This yellow reflex in these cases is caused by a cyclitic membrane behind the lens, the retina being also often detached. More minute investigation will frequently demonstrate signs of old iridocyclitis—posterior synechiæ, deep anterior chamber, pigment on the lens capsule, softening of the eye, &c. There can be little doubt that these cases are due to metastatic inflammation caused by pyogenic organisms, which have been incapable of setting up suppuration, and have gradually died out. Probably the natural resistance of the tissues in these young patients is greater than in the adult, a fact of which we have evidence in the behaviour of hypopyon ulcer in children.

It has already been said that tubercle of the optic nerve head is rare. The same is true of gummatous deposits in this situation, though I can show you specimens of each. The possibility of their occurrence must, therefore, be borne in mind, for metastatic inflammatory deposits, other than those due to the tubercle bacillus or the spirochæta pallida, also occur on the disc. An excellent example of a staphylococcic abscess on the disc has been recorded by Holmes Spicer. Early this year I showed at the Ophthalmological Society[17] a case of swelling of the disc, with much white exudate and some œdema of the surrounding retina in an apparently perfectly healthy woman. There were signs here of partial obstruction of the central vein of the retina, and I am inclined to think that the condition was due to a metastatic inflammatory deposit. It is possible that some other atypical and obscure cases of thrombosis of the central vein have a similar inflammatory origin. I may cite another case, that of a girl, which I showed at the Ophthalmological Society last year.

All the conditions which I have briefly reviewed in the earlier part of my discourse are fairly straightforward and open to no doubt. They have taught us facts which it is impossible to refrain from utilising when we turn our thoughts to other obscure diseases of the eye. We do so the more readily because some of these diseases are unfortunately very common, whilst others, happily less common, are peculiarly disastrous in their results. As a type of the former group, I will take ordinary sub-acute or chronic iridocyclitis; of the latter, sympathetic ophthalmia.

Iridocyclitis is familiar to you as an extremely insidious disease leading to deterioration or even loss of sight in people who may otherwise appear to be quite healthy. I need not enter here into a description of its clinical features. Now, we know that iridocyclitis is often a result of gross metastatic inflammation, and we are thereby led to inquire whether the apparently idiopathic cases may not be due to a similar cause. In one well-defined group we are rewarded with success. There is no doubt that these are a sequel of pyorrhœa alveolaris. When the septic condition of the mouth is cured the cyclitis rapidly improves, and does not return. The causal connection of oral sepsis is unmistakable in these cases. Frequently, however, the mouth is in good condition. We then seek a septic focus in some other part of the body. It may be found in the nasal sinuses, in the generative organs (usually in women, who are frequently affected), or elsewhere. In many cases we fail entirely in our search. In these it is probable that the intestinal tract is at fault. Certainly many cases do well on calomel and other intestinal disinfectants. I have recently been adopting Metchnikoff's method of disinfection, displacing the bacterium coli by lactic acid bacilli, but I have not yet sufficient cases to authorise any statement as to results. In any case, the theory which best suits these cases is one of metastatic inflammation, either by bacterial invasion or by the circulation of free toxins. The part acted upon is the epithelium covering the processes of the ciliary body. In considering whether in this and other such diseases we are dealing with bacteria or their products, we must bear in mind that organisms like the diphtheria bacillus, which set free their toxins, are much less common than those whose toxic action is indissolubly bound up with their own vital activity. There remains of course the possibility of specific cytotoxins of non-bacterial origin, but these we may leave out of the question for the present. The subject is one which has appeared to me a likely field for experimental research, and I have recently been engaged in putting this view to the test. We possess an extremely delicate test of the very slightest disturbance of the secretory functions of the ciliary processes. If an animal is immunised against the red corpuscles of another animal of different species, the serum of the immunised animal contains specific hæmolysins, *i.e.* products in the serum which dissolve out the hæmoglobin from red corpuscles of the immunising animal. In such an experiment, if instead of blood serum the aqueous humour of the immunised animal is taken it is found that no hæmolysis occurs. Now it is well known that if the aqueous is drawn off and the anterior chamber is allowed to fill up again with freshly-secreted aqueous, the second aqueous is highly albuminous. The process of secretion of aqueous by the ciliary processes, though perhaps not simply an act of filtration from the blood, yet follows closely the laws of filtration. When the aqueous is drawn off the intraocular pressure sinks from about 25 mm. of mercury to the atmospheric pressure or zero. The difference of pressure on the two sides of the walls of the ciliary capillaries, intracapillary and extracapillary, is greatly increased. Hence the large molecules of the proteids of the blood plasma, which are dammed back under normal circumstances, now pass through the walls as constituents of lymph, or, as it is called in the case of the eye, the aqueous. Without going deeply into the theory of hæmolysis, it must be pointed out that the process depends upon certain products which are intimately associated with the proteid constituents of the blood plasma. It is not surprising, therefore, to find that the second aqueous, secreted under such circumstances as those described, contains the products necessary for hæmolysis if the animal has been previously immunised; in fact, it acts very much like the blood serum, though less strongly. Further, it has long been known that the aqueous in cases of iritis or iridocyclitis is highly albuminous. Under these circumstances also, in an immunised animal the aqueous contains the products essential for hæmolysis. The hæmolytic test is one of extraordinary delicacy, and it enables us to distinguish the very earliest signs of disturbance of the circulation in the ciliary body, such as is found in slight congestion or inflammation. I am at present applying the test to immune animals which have been inoculated intravenously with cultures or toxins of various pathogenic organisms. I think it not improbable that I may be able to discover a specific selectivity for the ciliary body amongst the various organisms.

Lastly, I will ask you to consider for a few moments the possibility of sympathetic ophthalmia being a form of metastatic inflammation. The disease is one which appeals strongly to all ophthalmologists, but it should appeal specially to Bristolians, for it is to a Bristol surgeon that we owe the most beneficent discovery in the history of this malign disease. I need scarcely tell you that I refer to Augustin Prichard, who in 1851 first taught that early excision of the exciting eye prevented the onset of the disease. Like all great discoveries, this treatment was received with distrust, even by that master of ophthalmology, von Græfe, but since it was the truth it had to prevail.

There are many theories of sympathetic ophthalmia, but one fact stands out clearly—it has all the characteristics of a disease due to the influence of bacteria. Attempts to discover the specific organism have hitherto failed, but the theory of a bacterial origin is not thereby invalidated. If it is accepted, how does the organism reach the sympathising eye ? It was natural that direct transmission by way of the optic nerves and chiasma should attract earliest attention, but I think that this so-called migratory theory must be abandoned. The bacterial metastatic theory, to my mind, best explains the facts of the case. It is probable that the organism undergoes development in the exciting eye, thus accounting for a latent period of from one to six weeks. It then enters the blood stream. It is only pathogenic to the eye, because only there does it find a suitable nidus, and even there only if the conditions—resistance of tissues, &c.—are favourable. Prolonged latent period is accounted for by encapsulation in the exciting eye, or in some rare cases in other organs of the body. It would require more time than I have at my disposal to enter fully into a discussion of the pros and cons of this theory of sympathetic ophthalmia, and it is the less necessary as I have dealt with the subject elsewhere.

### REFERENCES.

[1] Coats, *Royal London Ophth. Hosp. Reports*, xvi, 1905.
[2] Stock, *Klin. Monatsbl. f. Augenhkde.*, 1903, 1907; *Arch. f. Ophth.*, 1907.
[3] Poynton and Paine, *Trans. Ophth. Soc.*, 1903.
[4] Axenfeld, *Bacteriology of the Eye*, tr. by McNab, London, 1908.
[5] *Klin. Monatsbl. f. Augenhkde.*, 1903, Beilageheft.
[6] Silcock, Treacher Collins, *Trans. Ophth. Soc.*, 1900.
[7] Jessop, *Trans. Ophth. Soc.*, 1907.
[8] Paton, *Trans. Ophth. Soc.*, 1903.
[9] Parsons, *Trans. Ophth. Soc.*, 1904.
[10] Coats, *Royal London Ophth. Hosp. Reports*, xvii, 1908.
[11] Roth, *Virchow's Archiv.*, 1872,
[12] Axenfeld and Goh, *Arch. f. Ophth.*, xliii, 1897.
[13] Grunert, Inaugural Dissertation, Tübingen, 1902.
[14] Holmes Spicer, *Trans. Ophth. Soc.*, 1906, 1907.
[15] Schanz, *Zeitschrift f. Augenhkde.*, 1906, Beilageheheft.
[16] Pernet, *Berliner klin. Woch.*, 1904.
[17] Parsons, *Trans. Ophth. Soc.*, 1908.
For further references and information, see Parsons, *Pathology of the Eye*, vols. i to iv, London, 1904-8.

## Figures and Tables

**Fig. 1. Fig. 2. Fig. 3. f1:**